# Evaluation of Corrosion, Mechanical Properties and Hydrogen Embrittlement of Casing Pipe Steels with Different Microstructure

**DOI:** 10.3390/ma14247860

**Published:** 2021-12-18

**Authors:** Olha Zvirko, Oleksandr Tsyrulnyk, Sebastian Lipiec, Ihor Dzioba

**Affiliations:** 1 Department of Diagnostics of Materials Corrosion-Hydrogen Degradation, Karpenko Physico-Mechanical Institute of the NAS of Ukraine, 5 Naukova St., 79060 Lviv, Ukraine; otsyrulnyk@gmail.com; 2 Department of Machine Design, Faculty of Mechatronics and Mechanical Engineering, Kielce University of Technology, Av. 1000-an. of Polish State 7, 25-314 Kielce, Poland; slipiec@tu.kielce.pl (S.L.); pkmid@tu.kielce.pl (I.D.)

**Keywords:** casing pipe steel, microstructure, mechanical properties, corrosion resistance, hydrogen embrittlement, true stress–strain dependences, FEM modelling

## Abstract

In the research, the corrosion and mechanical properties, as well as susceptibility to hydrogen embrittlement, of two casing pipe steels were investigated in order to assess their serviceability in corrosive and hydrogenating environments under operation in oil and gas wells. Two carbon steels with different microstructures were tested: the medium carbon steel (MCS) with bainitic microstructure and the medium-high carbon steel (MHCS) with ferrite–pearlite microstructure. The results showed that the corrosion resistance of the MHCS in CO_2_-containing acid chloride solution, simulating formation water, was significantly lower than that of the MCS, which was associated with microstructure features. The higher strength MCS with the dispersed microstructure was less susceptible to hydrogen embrittlement under preliminary electrolytic hydrogenation than the lower strength MHCS with the coarse-grained microstructure. To estimate the embrittlement of steels, the method of the FEM load simulation of the specimens with cracks was used. The constitutive relations of the true stress–strain of the tested steels were defined. The stress and strain dependences in the crack tip were calculated. It was found that the MHCS was characterized by the lower plasticity on the stage of the neck formation of the specimen and the lower fracture toughness than the other one. The obtained results demonstrating the limitations of the usage of casing pipes made of the MHCS with the coarse-grained ferrite/pearlite microstructure in corrosive and hydrogenating environments were discussed.

## 1. Introduction

Steel casing pipes are the main mechanical structural barrier elements during oil and gas oilfield lifecycle and beyond. One of the main integrity issues of oil and gas wells is associated with casing damage [1,2]. Pipe steels produced by different manufactures, which met the requirements to strength and plasticity [3,4], can have a different microstructure [5,6,7,8]. A majority of them utilize a ferritic–pearlitic or ferritic–bainitic microstructure. These pipes are usually operated under the influence of both corrosive and hydrogenating environments and mechanical loading. The long-term impact of these factors on pipes leads to a significant decrease in the initial corrosion resistance and mechanical properties of steels [9,10,11,12,13], which can influence negatively on their serviceability.

Corrosion impacted significantly on the performance of casings [5,6,14,15,16]. At the stage of well arrangement, casings are subjected to drilling fluids. The working environments used during the fracturing of shale layers under operation of shale gas wells are also corrosive. During operation, casing is regularly exposed to corrosive chemical environments, which are used to remove deposits of salts and paraffins. Acidic solutions are the most often used for this purpose. Since hydrogen evolution is possible during electrochemical corrosion of a metal in acidic solutions, the susceptibility of a casing steel to hydrogen embrittlement, stress corrosion cracking, and corrosion fatigue is also important for assessing the integrity issues for casing [5,17].

Dissolved gases such as oxygen O_2_, carbon dioxide CO_2_, and hydrogen sulfide H_2_S have a decisive influence on the corrosivity of an environment [14,18]. It was demonstrated by the analysis of casing failures [5,19] that, even when casing pipes made of steels that meet the increased requirements for operation in sour wells were used, they were often fractured due to corrosion or hydrogen-induced cracking during operation in non-sour wells.

Casing pipes are constantly subjected to shock loads during the operation of wells as a result of the descent and ascent of internal equipment. Therefore, fracture toughness and impact strength are very important characteristics for assessing their performance [20,21]. The requirements to some mechanical properties (yield and ultimate strength, elongation, impact toughness, and hardness) of casing pipe steels depending on steel grade and application are specified in the regulatory documents [3,4]; however, there is no limitation concerning the steel microstructure. Nevertheless, a type of microstructure significantly affects the mechanical and corrosive properties of casing pipe steels [7,8,14,20,21,22,23], and their thermal-mechanical treatment is successfully used for performance improvement [7,8]. The recent design of steels has been focused on a bainitic microstructure in order to achieve higher strength, rather than that of a ferritic–pearlitic microstructure [20,21,23]. After analyzing numerous research data on the influence of a microstructure of carbon and low alloy steels on corrosion performance, the authors [14] concluded that it is impossible to draw out general regularities because of the complexity of the issue and the various influenced factors involved. Thus, the performance of carbon steel with seemingly the same microstructure produced according to the same standard, but produced by different manufacturers, can be different [14].

Nowadays, the modern methods employed to determine the current metal state and to predict the residual life-time include both experimental studies and computer modelling based on the finite element method (FEM). To obtain reliable results, it is necessary to define the true stress–strain dependences for analyzed materials. Based on the analysis of the obtained true stress–strain diagrams, it is possible to assess the condition of metal qualitatively [23,24]. The calculations enable the determination of the stress and strain dependencies at the crack tip and an estimation of the fracture toughness of the material. A method based on an FEM calculation using the true stress–strain diagrams has been effectively utilized in various aspects of assessing the mechanical behavior of structural materials, in particular, to study the operational degradation of pipe steels of gas transit pipelines [12,24] and casings [23].

In the present work, two casing pipe steels with different microstructures, from the point of view of their susceptibility to corrosion, hydrogen embrittlement, and brittle fracture, were investigated. The main objective of the study was to assess their serviceability in corrosive and hydrogenating environments under operation in an oil and gas oilfield. To estimate the embrittlement of steel, FEM calculations were used, taking into account the true stress–strain relationships.

## 2. Materials and Testing Methods

Two carbon steels of casing pipes that are used in oil and gas wells in Ukraine were investigated: the medium carbon steel (MCS, the grade 50 steel, Ukrainian code) and the medium–high carbon steel (MHCS, the grade 32H2 steel, Ukrainian code). Samples were taken from commercially available casing pipes produced in Ukraine. The chemical composition of the studied materials was determined by optical emission spectroscopy using a SPECTROMAXx spectrometer. Chemical composition of the MCS was as follows, mass. %: 0.35 C; 1.22 Mn; 0.26 Si; 0.135 Cr; 0.01 S; 0.020 P; 0.129 Cu; Fe—balance. The MHCS was characterized by chemical composition, mass. %: 0.53 C; 0.72 Mn; 0.25 Si; 0.046 Cr; 0.03 S; 0.023 P; 0.024 Cu; Fe—balance.

**Figure 1:**
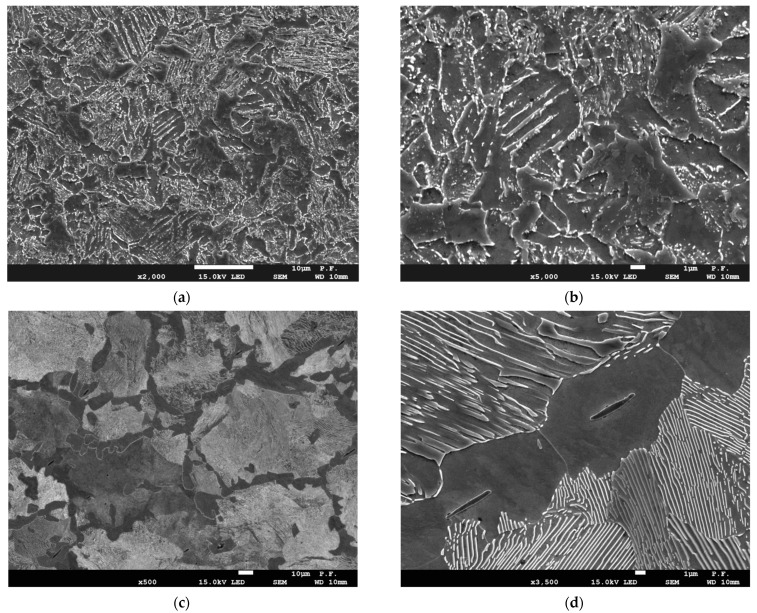
The SEM photographs of the steel microstructure: (**a**,**b**)—the MCS; (**c**,**d**)—the MHCS.

The scanning electron microscopy (SEM) was employed to observe the microstructures of the steels. The studied steels had the different microstructures, as shown in Figure 1. The microstructure of the MCS was bainite, with an average grain size of about 15 μm (Figure 1a,b). The MHCS is characterized by coarse-grained ferrite–pearlite microstructure with pearlite grains within a range of 30–100 μm in size, edged by ferrite layers of 5–15 μm wide (Figure 1c,d).

Specimens for tensile testing and impact toughness tests were cut out from commercially available casing pipes (outer diameter of 146 mm, wall thickness of 10.7 mm) in longitudinal direction, with the major axis parallel to the rolling direction of the pipe.

Basic mechanical properties of the investigated steels, namely modulus of elasticity *E*, lower yield point *σ*_YS_L_ and upper yield point *σ*_YS_H_, yield strength *σ*_Y_. ultimate strength *σ*_UTS_, elongation *δ*, and reduction in area (*RA*), were determined using uniaxial tensile tests of cylindrical specimens with an initial gauge length of 25 mm and a circular cross-section with a diameter of 5 mm. The strain rate was 3 × 10^−3^ s^−1^. Tensile testing of specimens was carried out in air at ambient temperature according to ASTM E8 standard [25] using a fully automated system of the test machine Zwick-100. The average values of strength and plasticity characteristics of the tested steels are presented in Table 1. Basic mechanical properties of the studied steels met the specifications they were supplied to.

For impact testing, the standard specimens 10 × 10 × 55 mm with V-type notch were machined from the casing pipes in the longitudinal direction and tested according to ASTM E23-07a standard [26]. V-notch was cut out from the internal surface of the casing pipes. The results are presented in Table 1.

Corrosion resistance of steels was evaluated by weight loss method according to ASTM G31 standard [27]. Immersion test followed by weight loss method to calculate the corrosion rate was used. The time of exposure was 1000 h. The flat steel specimens of thickness 1.5 mm were used. They were polished and rinsed in distilled water and in acetone before drying. The prepared specimens were stored in desiccators to avoid atmospheric corrosion before testing. Three parallel specimens were prepared to reduce measurement errors. The average corrosion rate of steels was determined.

Anodic dissolution rate of steels was investigated using BioLogic potentiostat by a potentiostatic method. A standard three-electrode electrochemical cell consisting of a working electrode made of the investigated steel, Ag/AgCl (saturated KCl) reference electrode, and auxiliary Pt electrode was used.

For the corrosive environment, simulating formation water in an oil and gas field, the following solution was used: 1% NaCl solution (pH = 7). For simulating acid formation water, the 1% NaCl solution was bubbled with CO_2_, and CH_3_COOH was added to achieve pH = 3.1. The test solutions were prepared from analytical grade reagents. The steels were tested under a temperature of 20 ± 2 °C.

Susceptibility of the investigated steels to hydrogen embrittlement was studied by tension of specimens in air after electrolytically pre-charging hydrogen. Cylindrical specimens with an initial gauge length of 25 mm and a circular cross-section with a diameter of 5 mm were used. The strain rate was 3 × 10^−4^ s^−1^. Hydrogen pre-charging of specimens was performed in an aqueous sulphuric acid solution with pH = 2 at current density *i* = 1.5 mA/cm^2^ for 2 h. The plasticity characteristics of the steels without and after hydrogenation from the point of view of their hydrogen embrittlement resistance were compared, and the susceptibility of the steels to hydrogen embrittlement was evaluated by a change in plasticity characteristics. With this purpose, the following parameters indicating loss in a certain plasticity characteristic were determined:

(1) $$\lambda_{\delta }=\frac{\delta -\delta_{H}}{\delta }\cdot 100\%;$$

(2) $$\lambda_{\mathrm{RA}}=\frac{RA-RA_{H}}{RA}\cdot 100\%,\;\;$$

where *δ*_H_ and *RA*_H_ are elongation and reduction in area of the specimens after hydrogenation, respectively.

Due to the insufficient thickness of the pipes for the production of standard specimens for fracture toughness testing, numerical FEM simulations of the loading of single-edged notch bend (SENB) specimens with the properties of the tested pipe materials were carried out. The simulation made it possible to calculate the stress and strain distributions in the front of the crack tip and, on the basis of these data, to qualitatively assess the crack resistance of the analyzed steels. An important aspect of performing these calculations is defining constitutional relationships of material true stress–strain dependencies. For this purpose, the method developed by Bai–Wierzbicki [28,29,30], with corrections introduced by Neimitz et al. [31,32], was used.

## 3. Experimental Results and Discussion 

### 3.1. Corrosion Resistance of Steels

**Figure 2:**
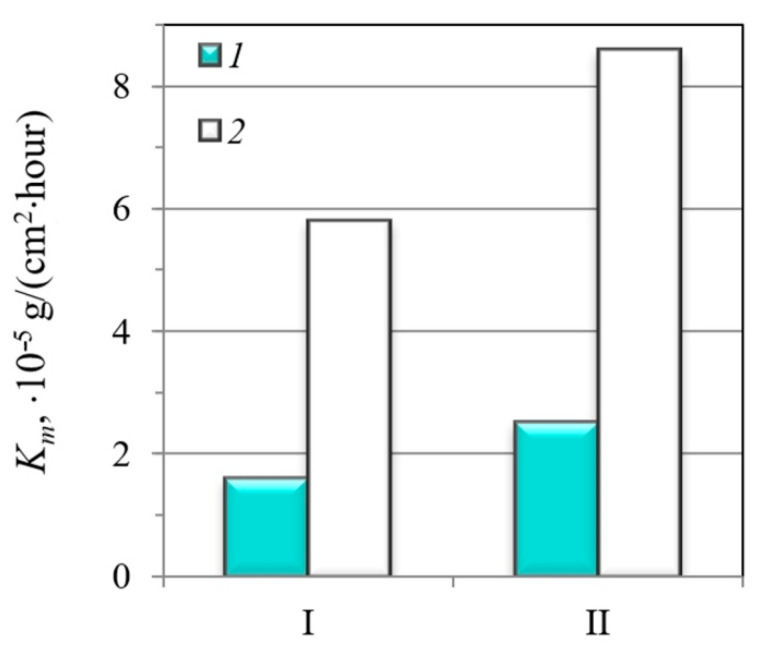
Corrosion rate of the MCS (I) and the MHCS (II) in 1% NaCl solution with pH = 7 (1) and 1% NaCl solution with CH_3_COOH (pH = 3.1), bubbled with CO_2_ (2). Time of exposure was 1000 h.

Figure 2 gives the results of the corrosion rate of the investigated steels in the 1% NaCl solution with different acidity, simulating neutral and acid formation water, determined by weight loss testing.

A significant difference between the corrosion resistances of the steels was revealed. Thus, the corrosion resistance of the MHCS is ~1.5 times lower than that of the MCS (Figure 2). Such a difference in the corrosion behavior was associated with the features of the microstructure; in particular, the MHCS had the microstructure with higher structural heterogeneity compared to that of the MCS: high ferrite/pearlite ratio, extended ferritic grains, and large pearlite grains, which caused its high electrochemical heterogeneity and formation of micro-galvanic couples. It was assumed that micro-galvanic couples formed between ferritic and pearlite grains drove the corrosion of the steel. Their activity, which determined the corrosion rate of the steels, depended on the intensity of the cathodic electrode reactions on the cathode areas. The active surface comprised local anodic and cathodic areas: the pearlite grains served as cathode and the nearby ferrite matrix suffers from a preferential anodic dissolution. Increasing cathodic area leads to the intensification of both cathodic electrode reactions and the anodic dissolution of ferrite. Therefore, the activity of micro-galvanic couples and, accordingly, the corrosion rate of the steel, increases with increasing grain sizes, which also means increasing the microstructural heterogeneity. Therefore, it can be concluded that the MHCS is susceptible to micro-galvanic corrosion when exposed to NaCl solution.

It should be noted that, in general, a high carbon steel corrodes faster than a low carbon steel in a CO_2_-containing acid environment, since cementite is a phase of low hydrogen overpotential. The effect of cementite as a cathodic region is pronounced in carbon steels with a carbon content of 0.15 mass % and higher [33]. Thus, the noticeably higher corrosion rate in API 5CT K55 steels compared to API 5CT P110 steels was revealed in the study [33], which was associated with a higher carbon content in K55 steels.

**Figure 3:**
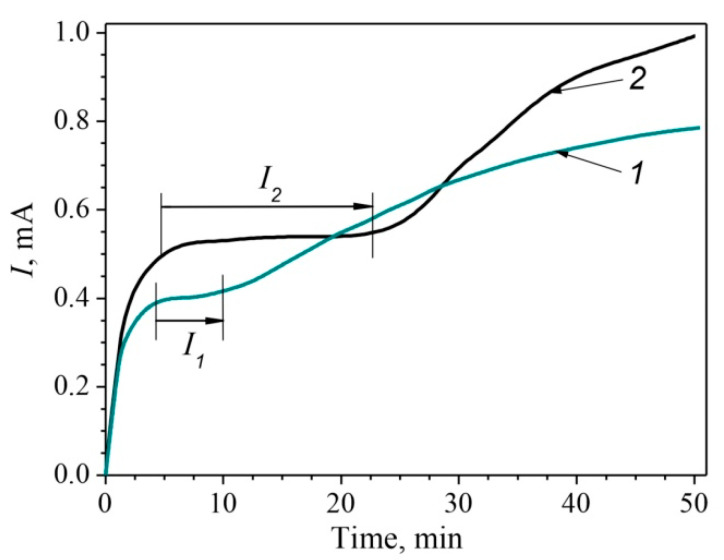
Current of anodic dissolution vs. time curves on the surfaces of the MCS (1) and the MHCS (2) in 1% NaCl solution with CH_3_COOH (pH = 3.1), bubbled with CO_2_, under anodic potential E_a_ = −0.1 V.

Current of anodic dissolution vs. time curves for the studied steels in 1% NaCl solution with CH_3_COOH (pH = 3.1), bubbled with CO_2_, under anodic potential E_a_ = −0.1 V, corresponding to active anodic dissolution, are presented in Figure 3. A plateau on chronoamperograms revealed at the initial stage of anodic dissolution for both steels (Figure 3) indicates the predominant dissolution of ferrite.

However, it should be noted that the MHCS is characterized by a slightly higher current value *I*_2_ on the plateau and, most importantly, a more extended plateau, which is obviously due to the higher ratio of free ferrite in its microstructure, compared with the other steel (*I*_1,_ Figure 3). The obtained data are consistent with those known for the selective corrosion of ferritic–pearlite steels in acidic media [34].

### 3.2. Susceptibility of Steels to Hydrogen Embrittlement

**Figure 4:**
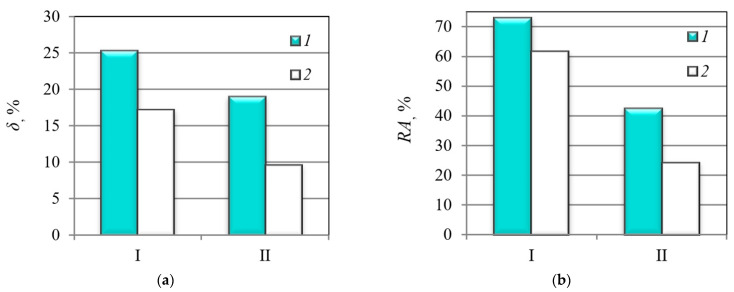
Elongation *δ* (**a**) and reduction in area *RA* (**b**) of the MCS (I) and the MHCS (II) determined by tensile testing: 1—without hydrogenation; 2—after electrolytic hydrogenation.

Tensile mechanical properties of the investigated casing pipe steels with different microstructures after their hydrogenation are presented in Table 2. It should be noted that the MCS was characterized by a higher plasticity compared to the MHCS (Figure 4) in both investigated states, uncharged and hydrogenated. This is probably due to the steel microstructure with highly dispersed bainite and the facilitation of multiple sliding at the stage of neck formation.

**Figure 5:**
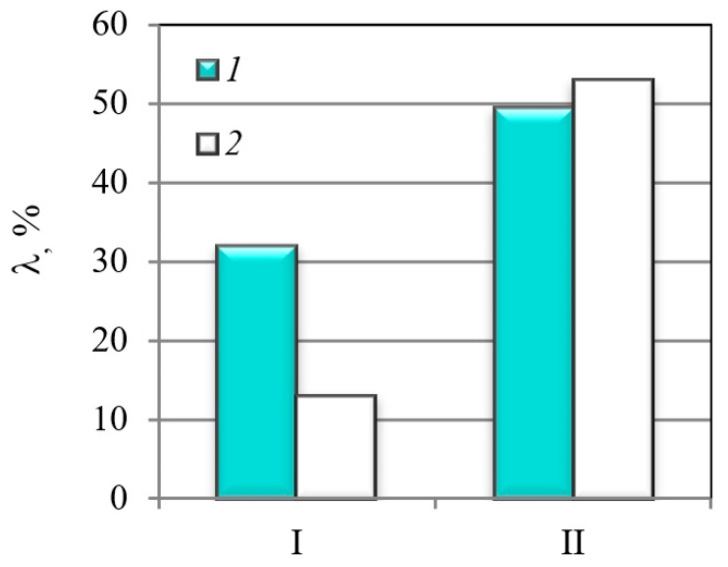
Relative changes in plasticity characteristics of the MCS (I) and the MHCS (II) caused by electrolytic hydrogenation: 1—λ_δ_; 2—λ_RA_.

The MHCS is characterized by higher susceptibility to hydrogen embrittlement than the MCS, as is demonstrated in Figure 5 by parameter λ_δ_ and *λ*_RA_, determined using Equations (1) and (2). These parameters indicate changes in the plasticity of steels due to pre-charging. Thus, the plasticity characteristics of the MHCS with lower strength and with coarse-grained microstructure (coarse-grained pearlite bordered by layers of ferrite) decreased almost two-fold as a result of the hydrogen embrittlement caused by preliminary hydrogen charging (Table 1 and Table 2). At the same time, the MCS with higher strength and higher initial characteristics of plasticity was less sensitive to hydrogen embrittlement, especially in terms of changes in reduction in area (Figure 5).

Hydrogen, which penetrates into a steel due to its interaction with corrosive media, is known to cause hydrogen embrittlement, accelerate crack growth, as well as facilitate deformation aging and degradation of steels [12,35,36]. The hydrogen/steel interaction is influenced by hydrogen penetration into steel and hydrogen localization. For hydrogenated metal, the role of temperature and operating stresses as factors in accelerating the diffusion of hydrogen increases. It is generally recognized that interfacial boundaries are the most effective traps for hydrogen [37]. According to [38], hydrogen during the strain aging of metals is localized mainly in dislocation traps (low-energy traps) inside the grains (in the atomic state), and stresses increase the ability of the metal to trap hydrogen [39]. Therefore, a steel with a higher dislocation density traps more hydrogen, which causes its higher susceptibility to hydrogen-induced cracking [40]. At the same time, hydrogen is localized in vacancies and their clusters [38,39] under the combined action of stresses and hydrogen, which leads to the increasing density of vacancies and their merging. Carbon has a significant influence on hydrogen trapping in steel, since it occupies lattice defects instead of hydrogen. Thus, with an increase in the carbon content in a steel from 6 ppm to 0.1%, the binding energy between dislocations and hydrogen decreases from 24.9 to 17.7 kJ/mol, while the portion of hydrogen in dislocation traps decreases [41]. Accordingly, hydrogen, on the one hand, accelerates the deformation, aging, and degradation of steels, and, on the other hand, facilitates intergranular brittle fracture by the mechanism of hydrogen embrittlement.

Therefore, assuming the same content of absorbed hydrogen in both investigated steels, in the MCS with a more dispersed bainite structure and, accordingly, with a larger area of interfacial and intergranular boundaries, hydrogen concentration at the boundaries would be lower than in the MHCS. In such a case, higher resistance of the MCS to hydrogen embrittlement could be explained by a lower hydrogen concentration at the interfacial and intergranular boundaries compared to the MHCS.

Thus, the existing standard and regulations for casing pipe steels [3,4] do not take into account the possibility of their brittle fracture under the action of corrosive and hydrogenating environments during operation; to ensure safe operation of oil and gas oilfield, the regulatory documents should be supplemented with requirements for resistance to hydrogen embrittlement for casing pipe steels.

### 3.3. Impact Toughness Testing

**Figure 6:**
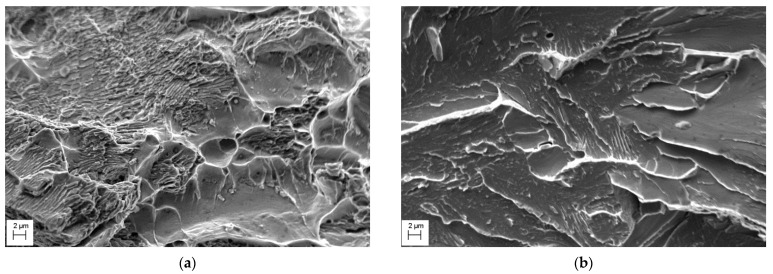
Fracture surfaces of the specimens made of the MCS (**a**) and MHCS (**b**) of casing pipes after impact toughness testing.

The impact strength values of the investigated steels show a remarkable difference. The impact toughness KCV of the MCS was 16.2 J/cm^2^. However, the lower strength MHCS was characterized by the very low value of impact strength KCV = 2.2 J/cm^2^; it absorbs about one-seventh of the impact energy absorbed by the specimen made of the higher strength MCS. The impact fracture surfaces of the studied steels are presented in Figure 6. The MCS specimen showed mostly ductile fracture with some brittle fragments on the ductile fracture surface (Figure 6a). The fracture surface of the MHCS predominantly exhibits the cleavage fracture mechanism (Figure 6b).

Since the characteristics of the resistance to brittle fracture of casing pipe steels with a low-strength grade are not regulated by normative documents [3,4], the low values of impact toughness are not formally the basis for making the decision that these steels are not suitable for operation. However, it is well known that steels with a low resistance to brittle fracture are particularly sensitive to the action of corrosive and hydrogenating environments. This factor is taken into account when analyzing the serviceability of oil and gas pipelines [10,11,12,13] and, accordingly, there is no reason to make an exception for casing pipes. The probability of the brittle fracture of casing pipes during operation is high due to the presence of shock loading, the effect of which is definitely enhanced by the deep corrosion damage of the inner surface of the casing pipes during their long-term operation.

### 3.4. FEM Assessment of the Brittle Fracture Tendency of Casing Steels

The material tendency of the tested pipes to crack according to the brittle mechanism was analyzed by simulating the load of three-point bending specimens with a crack—SENB, using FEM in the ABAQUS program. Numerical tests allow for the calculation of the values of stresses and strains in the material in the front of the crack tip and, on the basis of their distributions, to conclude about the material’s fracture toughness [42,43]. The reason for conducting numerical tests was also associated with the inability to make normative specimens for determining the *K*_IC_ or *J*_IC_ fracture toughness characteristics from the sections of the analyzed pipes due to insufficient pipe thickness.

Performing numerical calculations requires defining the constitutive relationship of the material—the stress–strain relationship in true values. In order to create the constitutive relationships of the material, the data from the uniaxial tensile tests of the specimens were used. The constitutive relationships of the materials were defined on the basis of the method proposed by Bai and Wierzbicki [28,29,30] with corrections introduced by Neimitz et al. [31,32]. According to this method, the stress function *σ_yld_* (Equation (3)) depending on the plastic strain, the stress triaxiality factor (STF) *ƞ* (Equation (4)), and the Lode parameter are presented in the following form [28,29,30]:

(3) $$\sigma_{yld}=\bar{\sigma }\left(\bar{\varepsilon_{pl}}\right)\left[1-c_{\eta }\left(\eta -\eta_{0}\right)\right]\left[c_{\theta }^{s}+\left(c_{\theta }^{ax}-c_{\theta }^{s}\right)\left(\gamma -\frac{\gamma^{m+1}}{m+1}\right)\right].$$

In Formula (3) *σ_yld_* is a function describing true stresses with respect to plastic strain obtained from experimental studies. The stress triaxiality factor *ƞ* is defined as:

(4) $$\eta =\frac{\sigma_{m}}{\sigma_{e}},$$

where *σ_m_*—medium stress, *σ_e_*—effective stress, *η*_0_—reference value of STF (for a smooth specimen in uniaxial tension—*η*_0_ = 0.33).

The function *γ* characterizes the curve that is located between the contours in the deviator plane, in the principal stress space. The function *γ* (Equations (5)–(10)) takes values within the limits 0–1, where 0 occurs for plane strain and shear, and 1—for axial symmetry. It is calculated from the following relation [28,29,30]:

(5) $$\gamma =\frac{cos\left(\frac{\pi }{6}\right)}{1-cos\left(\frac{\pi }{6}\right)}\left[\frac{1}{cos\left(\theta -\frac{\pi }{6}\right)}-1\right]=6,46\left[sec\left(\theta -\frac{\pi }{6}\right)-1\right],$$

where *θ*—the Lode angle lies within 0 ≤ *θ* ≤ π/3. The Lode angle is a function of the second and third invariants of the stress deviator:

(6) $$cos\left(3\theta \right)={\left(\frac{r}{\sigma_{e}}\right)}^{3}=\xi =\frac{27}{2}\frac{J_{3}}{\sigma_{e}^{3}};$$

(7) $$r={\left[\frac{27}{2}det\left(s_{ij}\right)\right]}^{\frac{1}{3}}={\left[\frac{27}{2}\left(\sigma_{1}-\sigma_{m}\right)\left(\sigma_{2}-\sigma_{m}\right)\left(\sigma_{3}-\sigma_{m}\right)\right]}^{\frac{1}{3}}$$

A normalized Lode angle, $\bar{\theta }$ , taking values between −1 and 1, is also used and described by the following relation:

(8) $$\bar{\theta }=1-\frac{6\theta }{\pi }=1-\frac{2}{\pi }arccos\xi .$$

The magnitude of the Lode parameter, *L*, can be represented by principal stress components $\sigma_{I},\;\sigma_{II},\;\sigma_{III}$ of the following form:

(9) $$L=-\frac{2\sigma_{II}-\sigma_{I}-\sigma_{III}}{\sigma_{I}-\sigma_{III}}.$$

There is a relationship between the values *ξ* and *L*:

(10) $$\xi =L\frac{9-L^{2}}{\sqrt{{\left(L^{2}+3\right)}^{3}}}.$$

The values *c_η_*, $c_{\theta }^{t}$, $c_{\theta }^{c}$,$\;c_{\theta }^{s}$, and m are determined experimentally. In Equation (3), the parameter $c_{\theta }^{ax}$, depending on the value of the Lode angle, (*θ*) is defined as: $c_{\theta }^{ax}$ = $c_{\theta }^{t}$ for *θ* ≥ 0 and $c_{\theta }^{ax}$ = $c_{\theta }^{c}$ for *θ* ≤ 0. For the stress–strain curve obtained experimentally using tensile test (cylindrical specimens): $c_{\theta }^{t}$ = 1; using compression test: $c_{\theta }^{c}$ = 1; using torsion test: $c_{\theta }^{s}$ = 1. A member with parameter m was added to the formula for the yield function, so that the yield surface is smooth and differentiable with respect to the Lode angle (*γ* = 1). The parameter m takes values mostly around unity.

Neimitz et al. [31,32,44,45] proposed a formula that allows for a very accurate calibration of the tensile curve at this stage of loading, which takes into account the effect of material softening. This phenomenon is identified with the process of initiation, development, and merging of voids in the material before the moment of destruction. A formula was proposed to calculate the magnitude of $c_{\eta }^{\prime }$ in the form [31]:

(11) $$c_{\eta }^{\prime }=c_{\eta }{\left[1+H\left(\varepsilon_{pl\_0}\right)\left(\varepsilon_{pl}-\varepsilon_{pl\_0}\right)\right]}^{\zeta },$$

where $\varepsilon_{pl\_0}$—the amount of plastic strain from the level where the softening effect on the tensile curve occurs (identified with the moment of void growth), H($\varepsilon_{pl\_0}$)—Heaviside function. The power exponent takes values greater than 1 (usually 5, 6).

**Figure 7:**
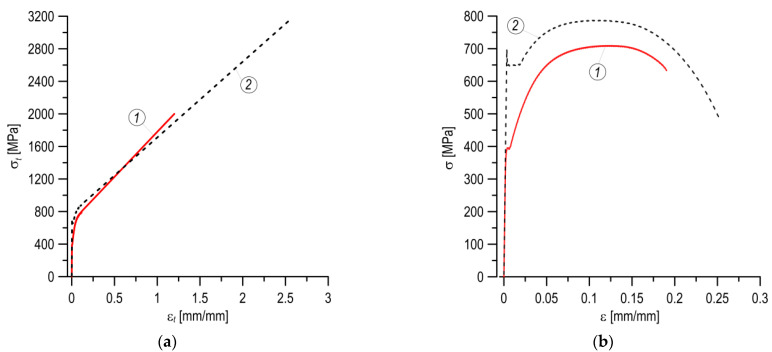
The stress–strain graphical relationships for the MHCS (1) and MCS (2) casing pipes: (**a**)—full true relationships; (**b**)—nominal curves.

As a result of the applied approach, described in detail in [32], constitutive relationships of the analyzed materials were obtained, the graphs of which are presented in Figure 7a.

The true stress–strain diagram for the MCS shows significantly higher strength characteristics compared to the MHCS. The MCS has a higher level of nominal characteristics: yield strength, ultimate strength, relative elongation, and relative reduction in area (Table 3, Figure 7b). The presented nominal tensile diagrams (Figure 7b) clearly show that a significantly higher plasticity is appropriate for the MCS, especially in the neck-forming section, which leads to a higher level of plastic strain values in the diagram in true values. For the MCS specimens, a much higher value of the relative reduction in area were also obtained, which leads to a high level of true stress. The critical values of the true strain and stress, *ε*_cr_ and *σ*_cr_, corresponding to the moment of specimen fracture, were determined from the true stress–strain curves (Figure 7a), and were presented in Table 3.

**Figure 8:**
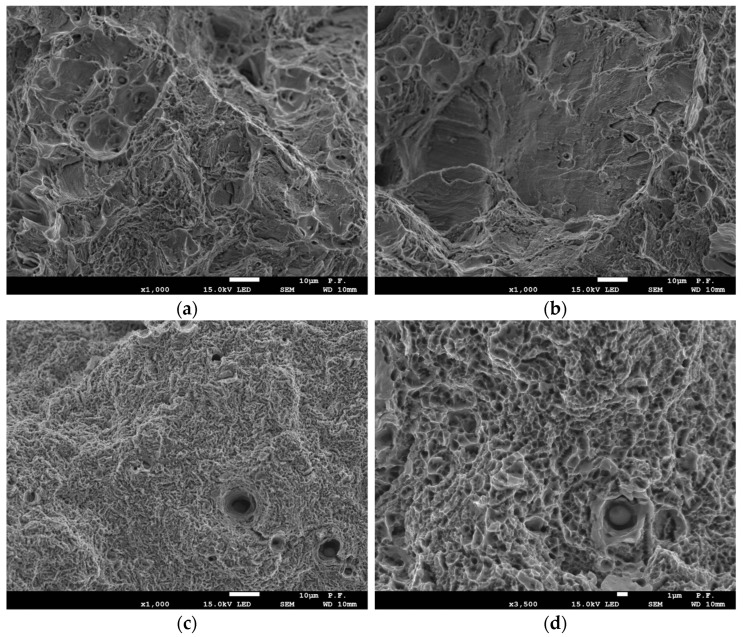
The fracture surfaces morphology of the specimens after tensile testing: (**a**,**b**)—MHCS; (**c**,**d**)—MCS.

Examination of the fracture surfaces carried out with the usage of SEM shows large differences in their morphology (Figure 8). The fracture surface obtained on the MHCS specimens has a complex structure (Figure 8a,b). There are mainly areas formed as a result of the growth and coalescence of voids. However, the voids in different areas have different sizes. It can be also noticed that there are relatively large areas on the fracture surface, where cracking was realized by sliding. Such a diverse morphology of the fracture surface is determined by the microstructure type of the MHCS. In the areas of perlite with different dispersibility of the plates, the cracking mechanism is realized through the development of voids, while, in the areas of ferrite, it is fractured by sliding.

The characteristic feature of the MCS is the fracture surface, which mainly consists of the small voids with diameters *d* < 1 µm (Figure 8c,d). Voids with larger dimensions (*d* > 1 µm) occur sporadically. This type of fracture is caused by the microstructure type, which consists of a fine-grained ferritic matrix and a large number of fine particles of the precipitates, which are almost evenly distributed.

**Figure 9:**
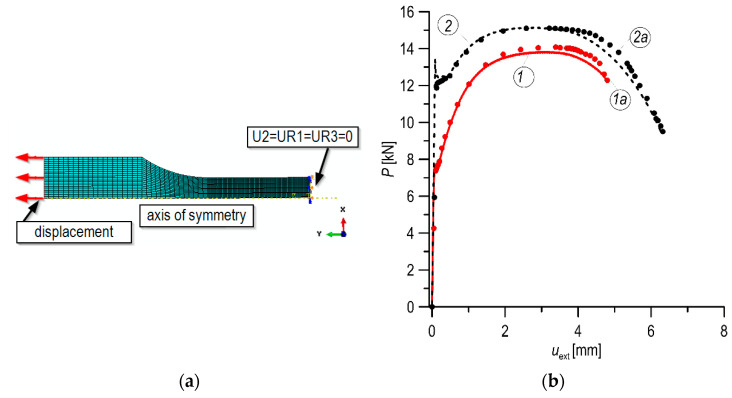
(**a**)—the FEM model of the tensile specimen; (**b**)—comparison of experimental (lines—1, 2) and numerically calculated (points—1a, 2a) curves of load–displacement of tensile specimens for the MHCS (1, 1a) and MCS (2, 2a).

The FEM model, conditions of boundary, and loading used for the tensile specimen are presented in Figure 9a (U2—displacement in direction y; UR1 and UR3—rotating around axes x and z). Experimentally and numerically calculated curves of the load–displacement of tensile specimens for the investigated steels were analyzed and compared, as shown in Figure 9b.

**Figure 10:**
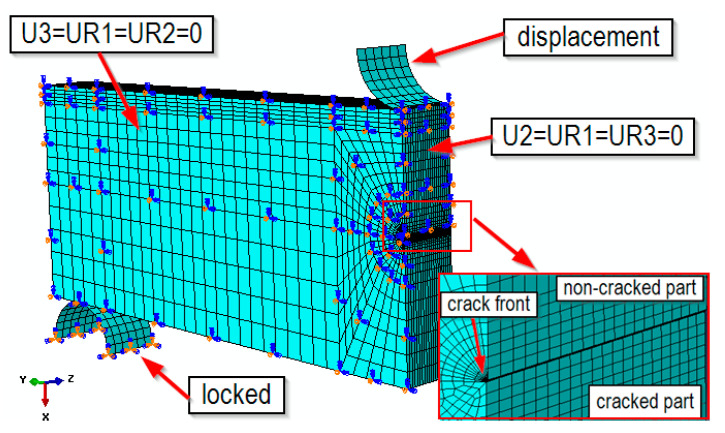
FEM model of the SENB specimen with boundary conditions.

In the next stage, a numerical simulation of the load on the three-point bending specimens with a one-sided notch, SENB, was carried out. Figure 10 shows the numerical model of the analyzed specimen and the boundary conditions imposed on the model. The numerical modeled specimen was loaded by the preset roller displacement. The stress and strain fields were analyzed for specimens under applied loading, when the measurement point displacement at the loading roll was equal to 1.2 mm.

According to the Ritchie–Knott–Rice criterion [46,47], brittle fracture occurs in a material if the level of stresses normal to the plane of the crack *σ*_22_ exceeds the critical one in the appropriate distance. Thus, the presence of a higher level of *σ*_22_ stresses in the material indicates a lower fracture toughness and a high probability of brittle fracture occurring therein. That is, the level of fracture toughness is closer to the lower plateau on the material’s brittle–plastic fracture transition curve. Since, in brittle cleavage cracking, the maximum values in the stress distributions occur directly at the crack tip, and with the increase in plasticity, the position of the maximum moves away from the crack tip [42,43], the maximum distance from the crack tip also qualitatively characterizes the material’s susceptibility to brittle cleavage fracture.

**Figure 11:**
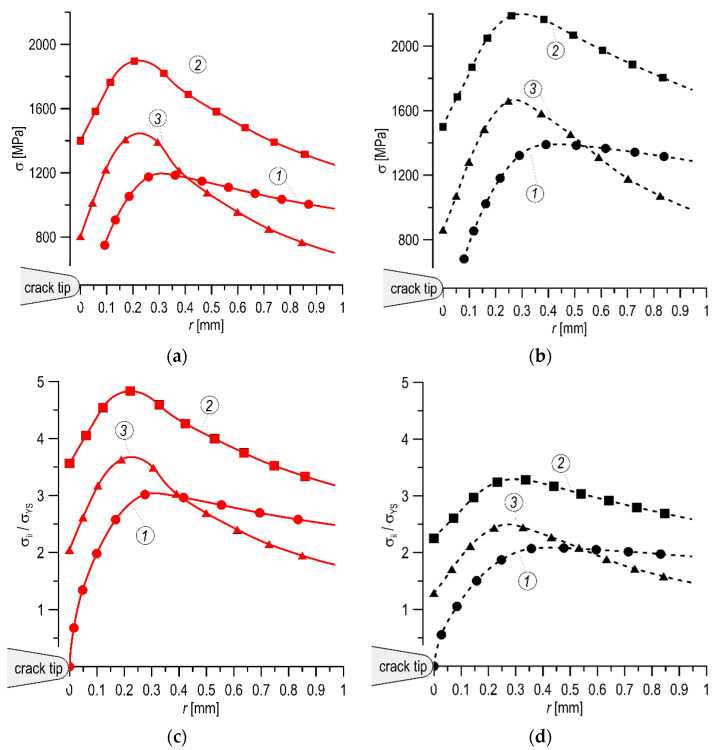
The graphs of stress distribution before the crack tip: of nominal value for the MHCS (**a**) and MCS (**b**) and of normalized by *σ*_YS_ for the MHCS (**c**) and MCS (**d**), where: 1—*σ*_11_; 2—*σ*_22_; 3—*σ*_33_.

The graphs of the stress components’ distribution before crack tip in the SENB specimens, obtained using calculations for the tested materials, are shown in Figure 11. The following stress components were calculated: *σ*_11_—in the direction of crack growth, *σ*_22_—in the direction perpendicular to the crack growth, *σ*_33_—in the direction of the specimen thickness. Stress component diagrams are presented in nominal values (Figure 11a,b) and as normalized by the value of the yield stress for the appropriate material (Figure 11c,d). When comparing the nominal diagrams, higher values of the stress components were observed for the MCS, which is obvious because this material is characterized by a higher yield strength. On the other hand, a comparison of the graphs in the normalized form allows one to properly assess the ability of these materials to occur with brittle cleavage fracture. In such a presentation, it is clearly and unequivocally visible that the relative level of the stress components in front of the crack tip in the MHCS is significantly higher than that in the MCS (Table 4). Moreover, the location of the maximum values of stress distributions for the MHCS is closer to the crack tip, which indicates a higher predisposition of the MHCS to brittle fracture in it.

**Figure 12:**
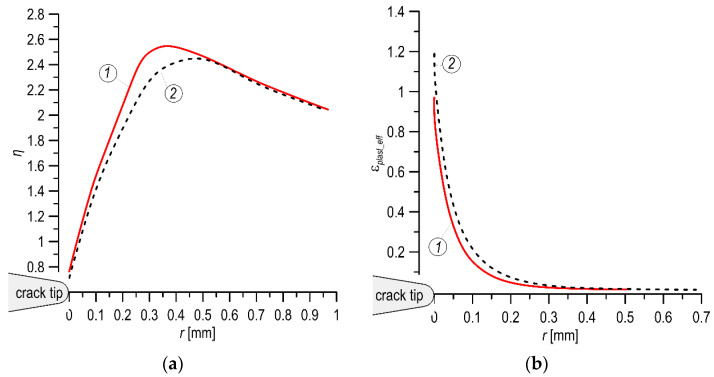
The STF (**a**) and the effective plastic strain (**b**) distributions for the MHCS (1) and MCS (2).

Similar tendencies in the distributions of the STF were observed for the studied steels (Figure 12). Thus, for the MHCS, the distribution reaches higher values, and the maximum value is closer to the crack tip (Figure 12a) than that for the other steel. For the MHCS, the level of effective plastic deformation immediately before the crack tip is lower than that in the MCS (Figure 12b, Table 4), which is also a feature of the material’s lower fracture toughness.

The presented results of experimental tests and numerical simulations clearly prove higher anti-corrosion and strength characteristics and also crack development resistance for the MCS compared to the MHCS. It is a very important fact that materials have a different microstructure type. The microstructure of the MHCS is coarse-grained ferrite-pearlite, while that of the MCS is fine bainite. The tests carried out on the 13CrMo4 steel used in power industry showed that the steel with fine-grained bainite microstructure had a high level of strength and fracture toughness characteristics [42,48]. On the other hand, for a steel of this type with a coarse-grained, ferritic–pearlitic microstructure, significantly lower values of strength and fracture toughness characteristics were obtained. Very beneficial strength and fracture toughness properties are also inherent to high strength steels, which also have a fine-grained bainitic microstructure, obtained as a result of thermomechanical treatment [49,50]. All these results indicate that low and medium carbon steels with a bainite microstructure have a very favorable set of mechanical and physical properties and can be successfully used as pipeline elements in oil and gas and energy industries.

## 4. Conclusions

The influence of microstructure features on corrosion, mechanical behavior, and resistance to hydrogen embrittlement has been studied experimentally for two casing pipe steels, and the true stress–strain relationships have been obtained using FEM calculations. The main conclusions are as follows:

The MHCS had microstructure consisted of coarse-grained pearlite edged by ferrite, the MCS had more dispersed bainite microstructure. The corrosion resistance of the MHCS in CO_2_-containing acid chloride solution, simulating formation water, was significantly lower than that of the MCS, which was associated with microstructure features. Coarse-grained microstructure of the MHCS led to high electrochemical heterogeneity and, consequently, to the intensification of corrosion.

The higher strength MCS with more dispersed microstructure was less susceptible to hydrogen embrittlement under preliminary electrolytic hydrogenation than the lower strength MHCS with coarse-grained microstructure.

The use of FEM numerical modeling in the process of loading the elements with a crack allows one to calculate the stress and strain distributions in front of the crack tip and, based on the obtained results, to evaluate the crack growth resistance of the material. Obtaining reliable results is based on the correct definition of the constitutive dependence of the material—the relationship of true stresses and strains. The FEM analysis carried out proves the higher tendency of the MHCS to the occurrence of brittle cleavage cracking.

The use of casing pipes made of the MHCS with a coarse-grained, ferrite/pearlite microstructure in corrosive and hydrogenating environments in oil and gas wells should be limited due to their high susceptibility to hydrogen embrittlement and brittle fracture.

## Figures and Tables

**Table 1:** Mechanical properties experimentally observed for the studied casing pipe steels.

Steel	*E* [GPa]	*σ*_YS_L_ [MPa]	*σ*_YS_H_ [MPa]	*σ*_UTS_ [MPa]	*δ* [%]	*RA* [%]	KCV [J/cm^2^]
MCS	209	654.0	697.3	786.9	25.3	72.9	16.2
MHCS	190	390.7	395.8	709.6	19.0	42.6	2.2

**Table 2:** Mechanical properties of casing pipe steels after their hydrogenation.

Steel	*σ*_Y_ [MPa]	*σ*_UTS_ [MPa]	*δ*_H_ [%]	*RA*_H_ [%]
MCS	652	773	17.2	61.6
MHCS	383	715	9.6	24.3

**Table 3:** The true strength and strain properties used to define the constitutive relationships for the casing steels.

Steel	*E* [GPa]	*σ*_YS_L_ [MPa]	*σ*_YS_H_ [MPa]	*ε*_UTS_ [mm/mm]	*σ*_UTS_ [MPa]	*ε*_cr_ [mm/mm]	*σ*_cr_ [MPa]
MCS	210	667.5	700.16	0.120	884.59	2.60	3160.72
MHCS	191	393.00	398.12	0.115	795.80	1.20	1956.71

where *ε*_UTS_—strain at ultimate tensile strength.

**Table 4:** The FEM calculated max values of stress components, STF, and effective plastic strain.

Steel	Max *σ*_11_ [MPa]	$\frac{\sigma_{11}}{\sigma_{YS\_L}}\;$	*r* [mm]	Max *σ*_22_ [MPa]	$\frac{\sigma_{22}}{\sigma_{YS\_L}}\;$	*r* [mm]	Max *σ*_33_ [MPa]	$\frac{\sigma_{33}}{\sigma_{YS\_L}}\;$	*r* [mm]	Max *η*	*r* [mm]	Max *ε*_ef.pl._ [mm/mm]	*r* [mm]
MCS	1355.4	2.0	0.49	2156.4	3.2	0.27	1644.4	2.4	0.27	2.4	0.49	1.15	0.01
MHCS	1205.7	3.0	0.35	1902.7	4.8	0.22	1445.3	3.6	0.21	2.5	0.35	0.95	0.01

## Data Availability

Data available on the request to the correspondence author.
